# The Bacilus of Tuberculosis

**Published:** 1882-06

**Authors:** 


### THE BACILLUS OF TUBERCULOSIS.

Since the publication of a paper by Aufrecht, in which he asserted that the centre of the miliary tubercle does not consist, as is generally believed, of detritus, but is filled by microscopical organisms, Baumgarten, at Konigsberg, and Koch, at Berlin, have almost simultaneously made public the results of their investigations in this direction. The carefully conducted experiments of Koch have been given additional publicity, and have been invested with a wide-spread popular enthusiasm by a letter, published in the *London Times*, from Professor Tyndall, in which he maintains the genuineness of what he calls Koch's discovery, and wherein he attests its importance in a scientific point of view.

The reputation which Koch enjoys as a cautious and painstaking investigator entitles his allegations to more attentive consideration than can with safety be accorded the brilliant but often evanescent discoveries, so-called, of many diligent microscopists; and, as we understand this question, there is a certain, perhaps an encouraging degree of unanimity concerning this discovery, on the part of several gentlemen; but, while Koch seems to have found his bacillus imbedded in the centre of the tubercle, Baumgarten's bacteria are found, not alone in the centre of the tubercle, but abounding also over its entire periphery.

However, we are not disposed to quibble over trifles; neither have we the temerity to suggest, in the face of apparently faultlessly conducted experiments, the possibility of mistaking an effect for the cause—of confounding a product of retrograde metamorphosis with the cause of destructive processes—and whatever the ultimate result of these possible triumphs may be, and whatever of practical benefit may ensue, one thing, at least, is certain, the alleged discovery has caused a very vigorous ripple in scientific circles and has excited a remarkable amount of popular attention.

Should the claims of its renowned author be confirmed, the fruit of his assiduous labors constitutes the first step in the great search after a cure for consumption. This step taken with safety, it remains for the enterprising therapeutist to discover a parasiticide that will surely demolish this vile intruder. An agent capable of penetrating the stony fortress in which the invader lies intrenched. A remedy, while it must be harmless to the patient, shall deal death to the destroyer. A drug possessed of such wonderful elective affinity that it shall pass without injury through the delicate tissues of the respiratory organs and seize with unrelenting severity and unfailing certainty the lilliputian giant which noiselessly but ceaselessly weaves its numberless enchaining cords around its unsuspecting and alas, its helpless victim. A substance which ferret-like can penetrate the inmost recesses inhabited by this most modern bug and with unerring precision annihilate the minute, rod-shaped demon.

But, levity aside, we shall await with eager expectancy further developments in this important and interesting field of inquiry, and can meanwhile only hope that our present incredulity will, ere long, be compelled to surrender unconditionally to an invincible array of established facts, and that the name of Koch with that of Jenner, illustrious, immortal; the greatest benefactors who ever lived—will be handed down in grateful reverence from generation to generation until time shall be no more.

